# Supplementary material for: Construction of an immunoinformatics-based multi-epitope vaccine candidate targeting Kyasanur forest disease virus
Source: PeerJ. 2025 Mar 21;13:e18982. doi: 10.7717/peerj.18982 (PMC11932114; doi:10.7717/peerj.18982)
Supplement: Supplemental Information 1 [file peerj-13-18982-s001.docx]

**Supplementary Figures and Tables**

**List of supplementary Figures**

**Supplementary Figure 1: Root-to-tip divergence of KFDV isolates based on complete genome**

**Supplementary Figure 2:** Left panel is the model generated by I-TASSER where [A] is the ribbon model [B] is the Ramachandran Plot [C] ProsaII Z-score plot. Right panel is the model after refinement where [A] is the ribbon model [B] is the Ramachandran Plot [C] ProsaII Z-score plot. [D] Predicted Secondary structure plot for vaccine construct.

**Supplementary Figure 3a:** Distance plot between amino acid residues belonging to vaccine construct and TLR2. Two interactions viz Glu382 (TLR2) with His115 (Vaccine construct) and Arg496 (TLR2) with Asp207 (Vaccine construct) are depicted in black and red colors respectively.

**Supplementary Figure 3b**: Distance plot between amino acid residues belonging to vaccine construct and TLR6. Two interactions viz Asn8 (TLR6) with Ser66 (vaccine construct) and Glu228 (TLR6) and Thr124 (vaccine construct) are depicted in black and red colors respectively.

**Supplementary Figure 4**: RMSD of the vaccine construct with TLR2-TLR6 docked complex. RMSD of the entire complex (black), vaccine construct (red), TLR2 receptor (green) and TLR6 receptor (blue). A) Run2 B) Run3

**Supplementary Figure 5**: RMSF of the docked vaccine construct. A) Replicate 2 B) Replicate 3

**Supplementary Figure 6:** Interaction plot for all the hydrogen bonds and Van der Waal’s interactions in the docked complex of vaccine construct with TLR2 at different time frames of simulations.

**Supplementary Figure 7:** Interaction plot for all the hydrogen bonds and Van der Waal’s interactions in the docked complex of vaccine construct with TLR6 at different time frames of simulations.

**Supplementary Figure 8**: Free energy of binding between vaccine construct and TLR2-TLR6 complex. TLR2 and TLR6 (black), vaccine construct and TLR2-TLR6 complex (red), vaccine construct and TLR2 (green), vaccine construct and TLR6 (blue). A) Replicate 2 B) Replicate 3**.**

**List of supplementary Tables**

**Supplementary Table 1: Details of 74 KFDV isolates used for phylogenetic analysis.**

**Supplementary Table 2:** Representative isolates identified based on the lineages observed in the phylogenetic tree**.**

**Supplementary Table 3:** Computational prediction of IL-4 and IL-10 stimulation by short-listed MHC-Class II epitopes belonging to envelope protein of KFDV

**Supplementary Table 4:** List of hydrogen and Van der Waal’s interacting residues between TLR2 receptor and vaccine construct

**Supplementary Table 5:** List of hydrogen and Van der Waal’s interacting residues between TLR6 receptor and vaccine construct

**Supplementary Figures**

**Supplementary Figure 1: Root-to-tip divergence of KFDV isolates based on complete genome**

**
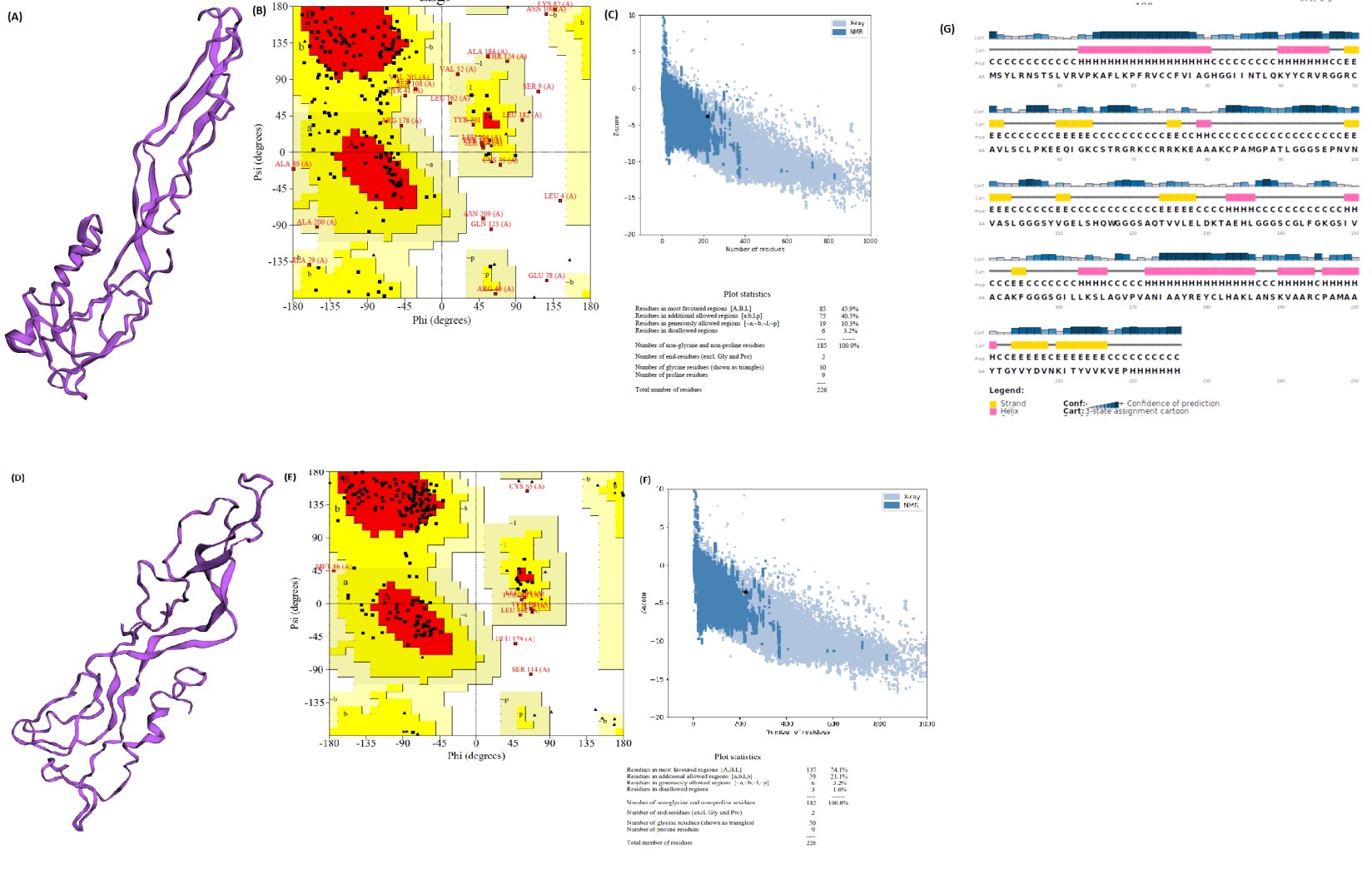
**

**Supplementary Figure 2:** Upper panel is the model generated by I-TASSER where [A] is the ribbon model [B] Ramachandran Plot [C] ProsaII Z-score plot.

Lower panel is refined model [D] Ribbon model [E] Ramachandran Plot [F] ProsaII Z-score plot

[G] Predicted secondary structure plot of vaccine construct.

**
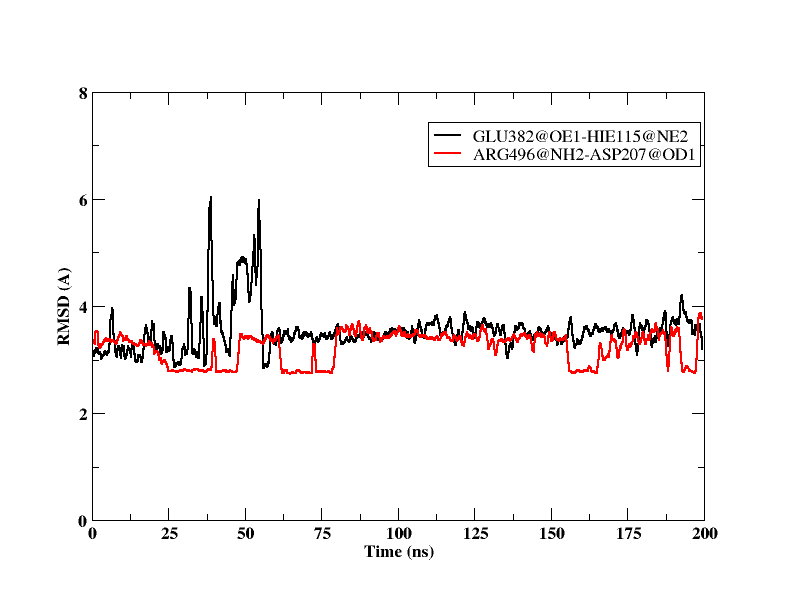
**

**Supplementary Figure 3a:** Distance plot between amino acid residues belonging to vaccine construct and TLR2. Two interactions viz Glu382 (TLR2) with His115 (Vaccine construct) and Arg496 (TLR2) with Asp207(Vaccine construct) are depicted in black and red colors respectively.

**
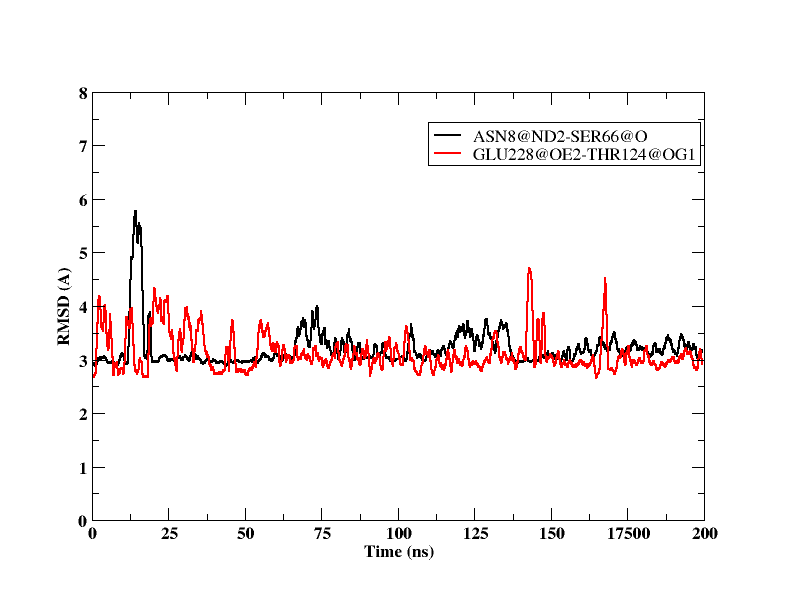
**

**Supplementary Figure 3b:** Distance plot between amino acid residues belonging to vaccine construct and TLR6. Two interactions viz Asn8 (TLR6) with Ser66 (vaccine construct) and Glu228 (TLR6) and Thr124 (vaccine construct).
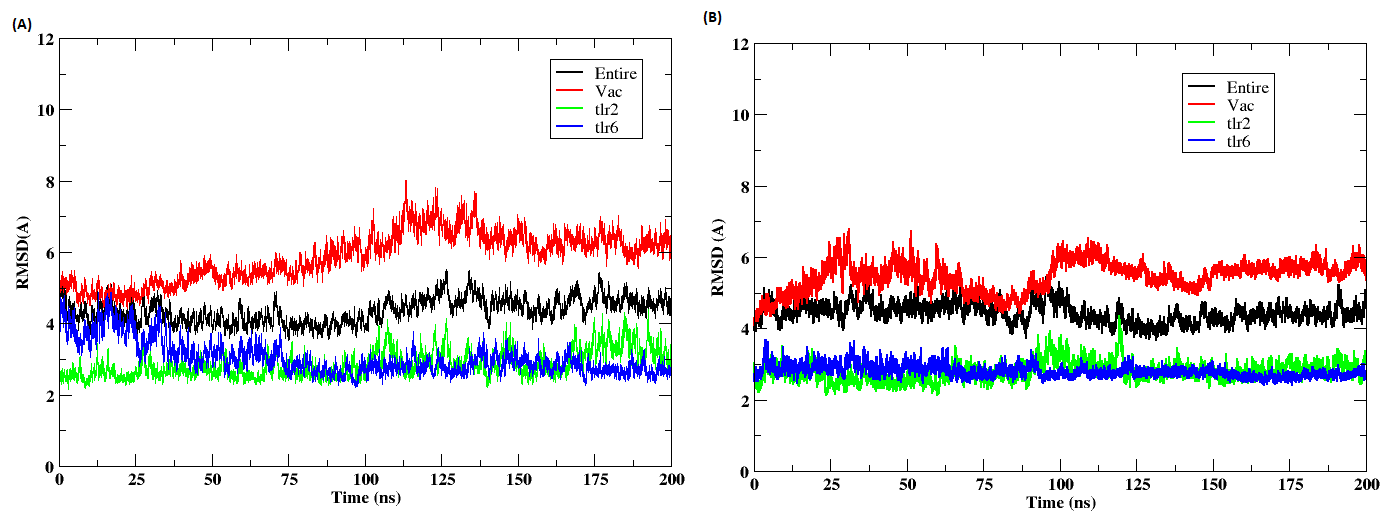


**Supplementary Figure 4:** RMSD of the vaccine construct with TLR2-TLR6 docked complex. RMSD of the entire complex (black), vaccine construct (red), TLR2 receptor (green) and TLR6 receptor (blue). A) Replicate 2 B) Replicate 3


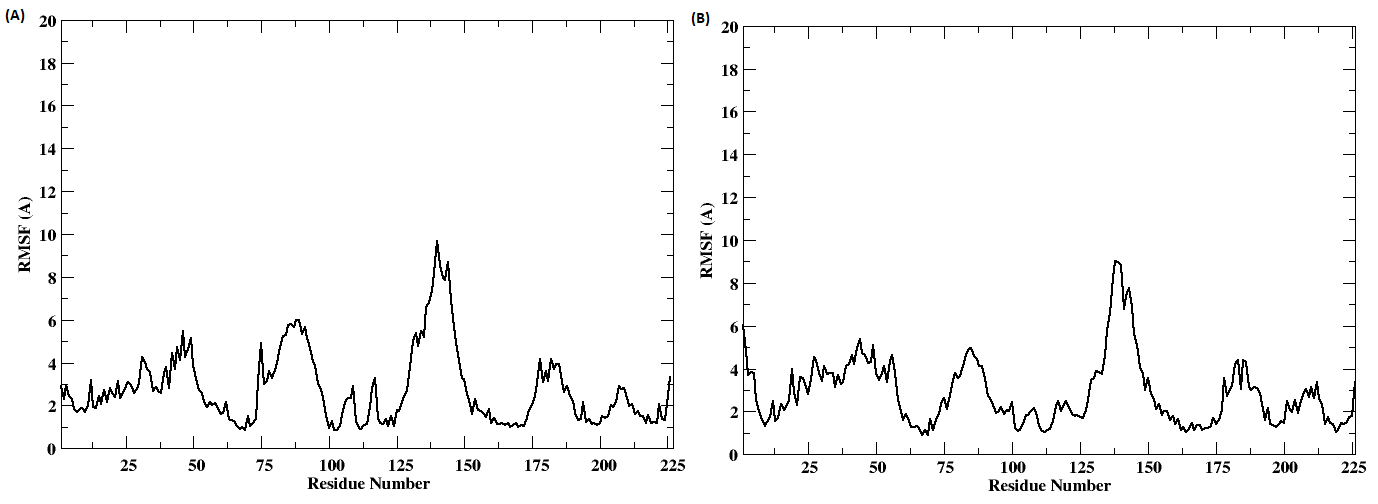


**Supplementary Figure 5:** RMSF of the docked vaccine construct. A) Replicate 2 B) Replicate 3

| *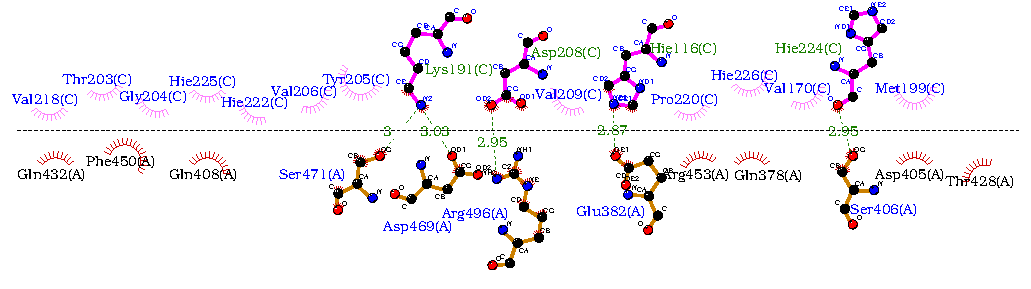*  *50ns* |
| --- |
| *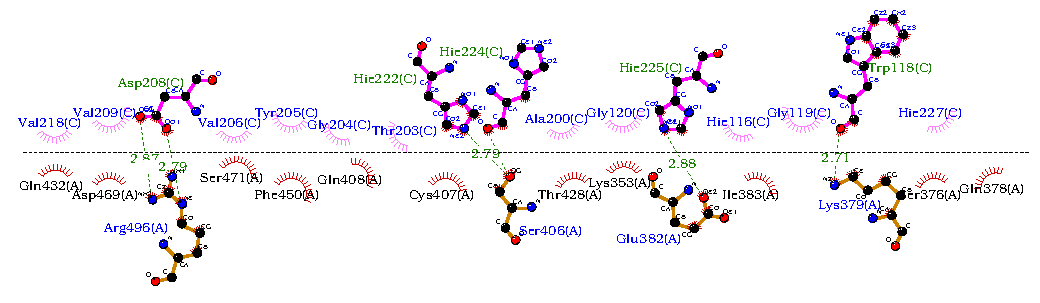*  *100ns* |
| *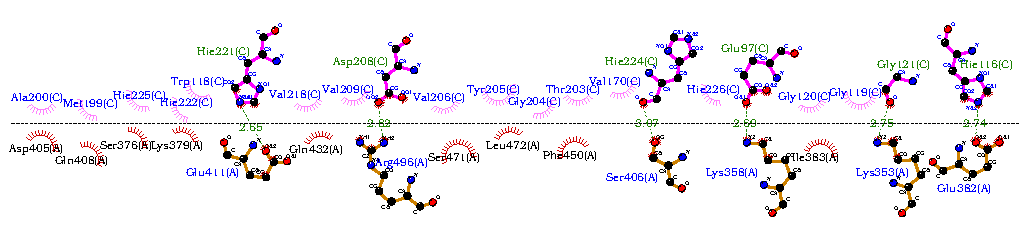*  *150ns* |
| *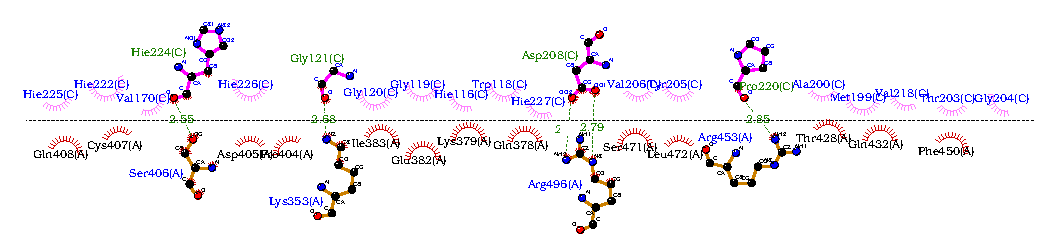200ns* |

**Supplementary Figure 6**: Interaction plot for all the hydrogen bonds and Van der Waal’s interactions in the docked complex of vaccine construct with TLR2 at different time frames of simulations.

| *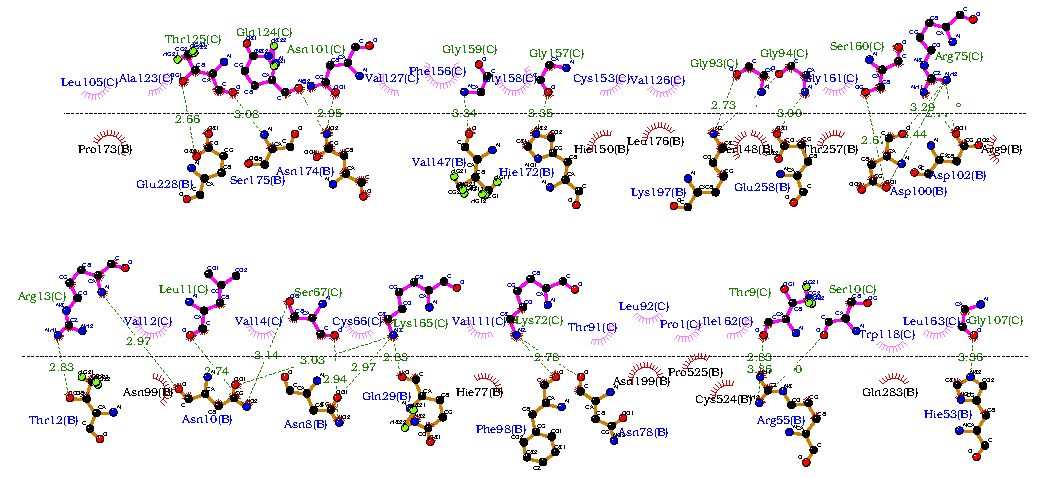*  *50ns* |
| --- |
| *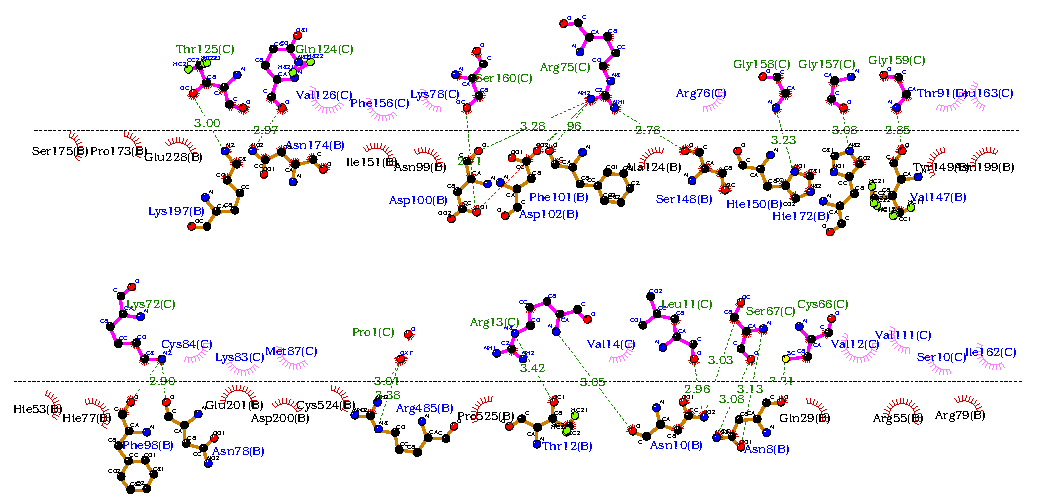*  *100ns* |
| *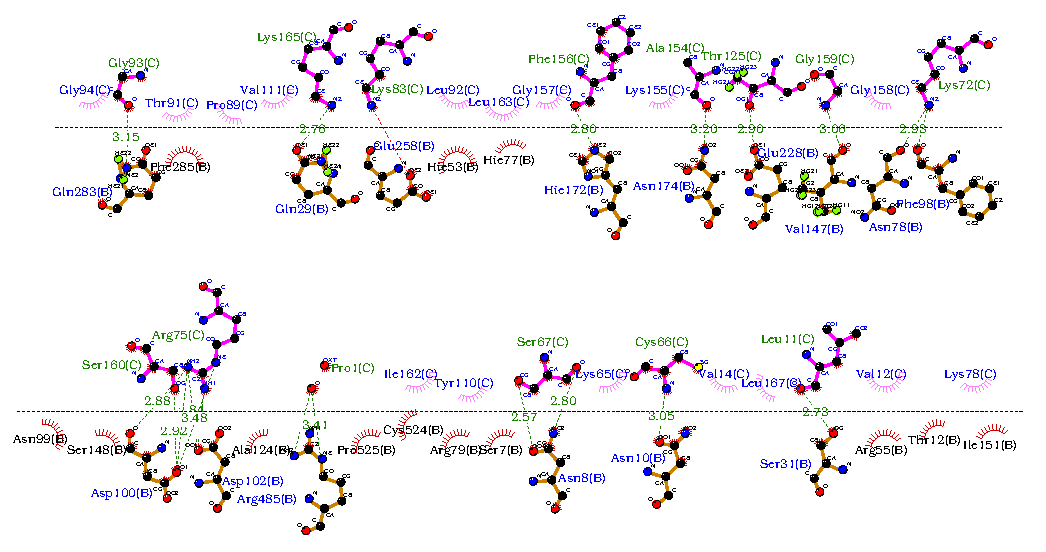*  *150ns* |
| *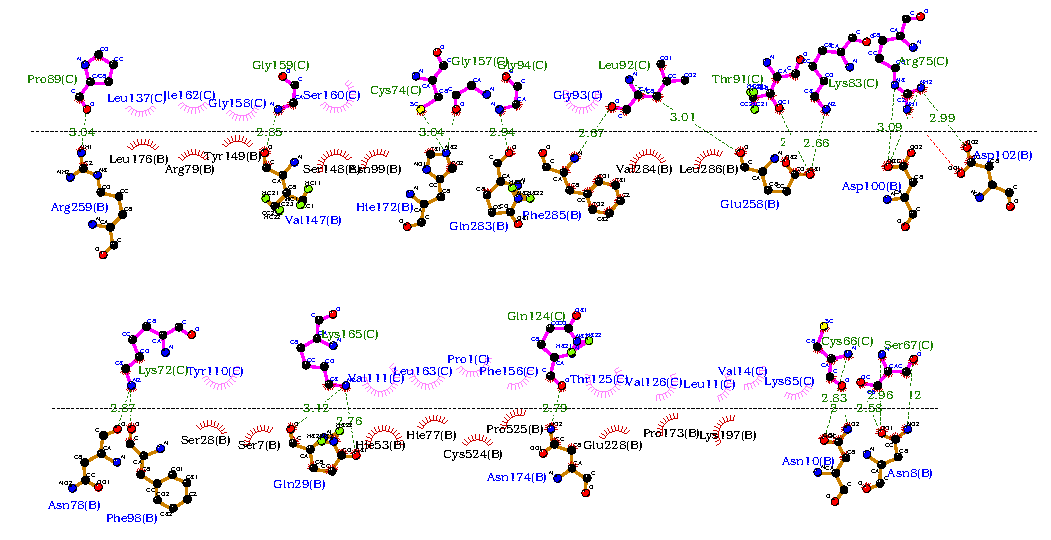200ns* |

**Supplementary Figure 7**: Interaction plot for all the hydrogen bonds and Van der Waal’s interactions in the docked complex of vaccine construct with TLR6 at different time frames of simulations.


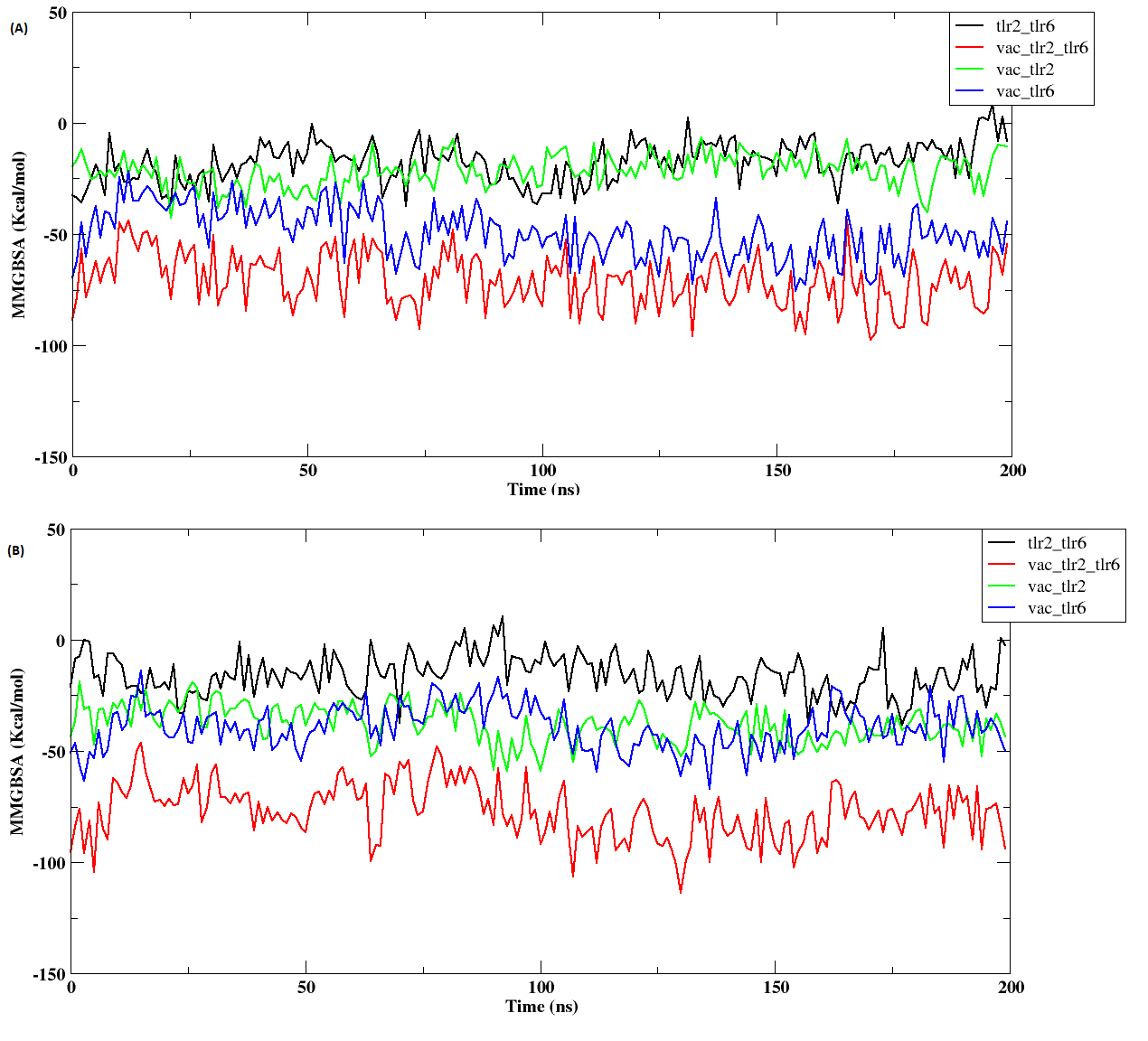


**Supplementary Figure 8:** Free energy of binding between vaccine construct and TLR2-TLR6 complex. TLR2 and TLR6 (black), vaccine construct and TLR2-TLR6 complex (red), vaccine construct and TLR2 (green), vaccine construct and TLR6 (blue). A) Replicate 2 B) Replicate 3

**Supplementary Tables**

**Supplementary Table 1: Details of 74 KFDV isolates used for phylogenetic analysis**

| **Sr.No** | **Sequence title in alignment** | **Accession ID** | **Source for Isolation (Human/Tick/Mouse)** | **Year of Isolation** | **Location** |
| --- | --- | --- | --- | --- | --- |
| 1 | P9605_P1-KA-H-1957 | OR179912 | Homo Sapiens | 1957 | India: Shimoga, Karnataka |
| 2 | MG720088-NIV_P9605-KA-H-1957 | MG720088 | Homo Sapiens | 1957 | India: Shimoga, Karnataka |
| 3 | MG720077-NIV_W1930-KA-M-1958 | MG720077 | Semnopithecus entellus | 1958 | India: Chimnoor, Karnataka |
| 4 | MG720090-NIV_G27678-KA-T-1959 | MG720090 | Haemaphysalis | 1959 | India: Kopalgadde, Karnataka |
| 5 | MG720099-NIV_W6043-KA-M-1959 | MG720099 | Semnopithecus entellus | 1959 | India: Belisiri, Karnataka |
| 6 | MG720112-NIV_601203-KA-H-1960 | MG720112 | Homo Sapiens | 1960 | India: Tudikoppa, Karnataka |
| 7 | MG720089-NIV_62957-KA-H-1962 | MG720089 | Homo Sapiens | 1962 | India: Gadag, Karnataka |
| 8 | MG720093-NIV_62844-KA-T-1962 | MG720093 | Haemaphysalis spinigera | 1962 | India: Gadag, Karnataka |
| 9 | MG720094-NIV_63696-KA-M-1963 | MG720094 | Semnopithecus entellus | 1963 | India: Shimoga, Karnataka |
| 10 | MG720084-NIV_642034-KA-T-1964 | MG720084 | Haemaphysalis turturis | 1964 | India: Shimoga, Karnataka |
| 11 | MG720109-NIV_652980-KA-T-1965 | MG720109 | Haemaphysalis | 1965 | India: Shimoga, Karnataka |
| 12 | MG720095-NIV_66364-KA-H-1966 | MG720095 | Homo Sapiens | 1966 | India: Sagar, Karnataka |
| 13 | MG720078-NIV_671004-KA-M-1967 | MG720078 | Semnopithecus entellus | 1967 | India: Dharwad, Karnataka |
| 14 | MG720097-NIV_67965-KA-H-1967 | MG720097 | Homo Sapiens | 1967 | India: Sagar, Karnataka |
| 15 | MG720100-NIV_71580-KA-T-1971 | MG720100 | Haemaphysalis spinigera | 1971 | India: Gunjnur, Karnataka |
| 16 | MG720107-NIV_72827-KA-M-1972 | MG720107 | Semnopithecus entellus | 1972 | India: Holekoppa, Karnataka |
| 17 | MG720098-NIV_A106-KA-H-2006 | MG720098 | Homo Sapiens | 2006 | India: Chikballapura, Karnataka |
| 18 | NIV12839_P1-KA-H-2012 | PP098210 | Homo Sapiens | 2012 | India: Shimoga, Karnataka |
| 19 | MG720079-NIV_121863-KA-H-2012 | MG720079 | Homo Sapiens | 2012 | India: Shimoga, Karnataka |
| 21 | MG720083-NIV_12869-KA-H-2012 | MG720083 | Homo Sapiens | 2012 | India: Shimoga, Karnataka |
| 22 | MG720103-NIV_12834-KA-H-2012 | MG720103 | Homo Sapiens | 2012 | India: Shimoga, Karnataka |
| 23 | MG720108-NIV_121865-KA-H-2012 | MG720108 | Homo Sapiens | 2012 | India: Shimoga, Karnataka |
| 24 | MG720081-NIV_131152-KA-H-2013 | MG720081 | Homo Sapiens | 2013 | India: Shimoga, Karnataka |
| 25 | MG720082-NIV_131151-KA-H-2013 | MG720082 | Homo Sapiens | 2013 | India: Shimoga, Karnataka |
| 26 | MG720085-NIV_AN131058-TN-M-2013 | MG720085 | Presbytes Entellus (Langur) | 2013 | India: Nilgiri, TamilNadu |
| 27 | MG720086-NIV_137459-KA-H-2013 | MG720086 | Homo Sapiens | 2013 | India: Shimoga, Karnataka |
| 28 | MG720087-NIV_134787-KA-H-2013 | MG720087 | Homo Sapiens | 2013 | India: Uttar kannada,Karnataka |
| 29 | MCL-14-H-344_NIV_142856_P1-KA-H-2014 | OR179910 | Homo Sapiens | 2014 | India: Shimoga, Karnataka |
| 30 | MCL-16-H-57_NIV_16877_P1-MH-H-2016 | OR179914 | Homo Sapiens | 2016 | India: Sindhudurg, Maharashtra |
| 31 | MCL-16-H-286_NIV_162235_P1-MH-H-2016 | OR179913 | Homo Sapiens | 2016 | India: Sindhudurg, Maharashtra |
| 32 | MG720091-NIV_164187(1297)-KA-H-2016 | MG720091 | Homo Sapiens | 2016 | India: Belgaum, Karnataka |
| 33 | MG720092-NIV_165058(1644)-KA-H-2016 | MG720092 | Homo Sapiens | 2016 | India: Belgaum, Karnataka |
| 34 | MG720096-NIVAN161919-GA-M-2016 | MG720096 | Semnopithecus entellus | 2016 | India: Caranzalem, Goa |
| 35 | MG720101-NIV16848-MH-H-2016 | MG720101 | Homo Sapiens | 2016 | India: Sindhudurg, Maharashtra |
| 36 | MG720104-NIV16827-MH-H-2016 | MG720104 | Homo Sapiens | 2016 | India: Sindhudurg, Maharashtra |
| 37 | MG720105-NIV16855-MH-H-2016 | MG720105 | Homo Sapiens | 2016 | India: Sindhudurg, Maharashtra |
| 38 | MG720106-NIV16877-MH-H-2016 | MG720106 | Homo Sapiens | 2016 | India: Sindhudurg, Maharashtra |
| 39 | MG720111-NIV162235-MH-H-2016 | MG720111 | Homo Sapiens | 2016 | India: Sindhudurg, Maharashtra |
| 40 | MG720115-MCL_16_T_79-MH-2016 | MG720115 | Ambliyomma | 2016 | India: Sindhudurg, Maharashtra |
| 41 | MG720118-MCL_16_T_343-MH-2016 | MG720118 | Haemaphysalis | 2016 | India: Sindhudurg, Maharashtra |
| 42 | MG720119-MCL_16_T_341-MH-2016 | MG720119 | Haemaphysalis | 2016 | India: Sindhudurg, Maharashtra |
| 43 | MG720120-MCL_16_T_363-KA-2016 | MG720120 | Haemaphysalis | 2016 | India: Shimoga, Karnataka |
| 44 | MG720122-MCL_16_T_346-MH-2016 | MG720122 | Haemaphysalis | 2016 | India: Sindhudurg, Maharashtra |
| 45 | MG720102-NIV_1721318-KA-H-2017 | MG720102 | Homo Sapiens | 2017 | India: Shimoga, Karnataka |
| 46 | MG720113-NIV_1721699-MH-M-2017 | MG720113 | Semnopithecus entellus | 2017 | India: Sindhudurg, Maharashtra |
| 47 | MG720114-NIV_1722297-KA-H-2017 | MG720114 | Homo Sapiens | 2017 | India: Shimoga, Karnataka |
| 48 | MG720116-MCL_17_T_296-GA-2017 | MG720116 | Haemaphysalis spinigera | 2017 | India: North Goa, Goa |
| 49 | MG720117-NIVAN1722825-GA-M-2017 | MG720117 | Black faced grey langur/Adult Male | 2017 | India: North Goa, Goa |
| 50 | MG720121-MCL_17_T_480-MH-2017 | MG720121 | Haemaphysalis spinigera | 2017 | India: Sindhudurg, Maharashtra |
| 51 | MCL-19-H-228-MH-H-2019 | OR162007 | Homo Sapiens | 2019 | India: Sindhudurg, Maharashtra |
| 52 | MCL-19-H-552-MH-H-2019 | OR162008 | Homo Sapiens | 2019 | India: Sindhudurg, Maharashtra |
| 53 | MCL-19-H-553-MH-H-2019 | OR162012 | Homo Sapiens | 2019 | India: Sindhudurg, Maharashtra |
| 54 | MCL-19-H-554-MH-H-2019 | OR162009 | Homo Sapiens | 2019 | India: Sindhudurg, Maharashtra |
| 55 | MCL-19-H-561-MH-H-2019 | OR162010 | Homo Sapiens | 2019 | India: Sindhudurg, Maharashtra |
| 56 | MCL-19-H-612-MH-H-2019 | OR162011 | Homo Sapiens | 2019 | India: Sindhudurg, Maharashtra |
| 57 | MCL-19-H-650-MH-H-2019 | OR162013 | Homo Sapiens | 2019 | India: Sindhudurg, Maharashtra |
| 58 | MCL-19-H-686-MH-H-2019 | OR162014 | Homo Sapiens | 2019 | India: Sindhudurg, Maharashtra |
| 59 | MCL-19-H-783-MH-H-2019 | OR162015 | Homo Sapiens | 2019 | India: Sindhudurg, Maharashtra |
| 60 | MCL-19-H-888-MH-H-2019 | OR162017 | Homo Sapiens | 2019 | India: Sindhudurg, Maharashtra |
| 61 | MCL-19-H-908-MH-H-2019 | OR162019 | Homo Sapiens | 2019 | India: Sindhudurg, Maharashtra |
| 62 | MCL-19-H-1180-MH-H-2019 | OR162020 | Homo Sapiens | 2019 | India: Sindhudurg, Maharashtra |
| 63 | MCL-19-H-689-MH-H-2019 | OR162016 | Homo Sapiens | 2019 | India: Sindhudurg, Maharashtra |
| 64 | MCL-19-H-897-MH-H-2019 | OR162018 | Homo Sapiens | 2019 | India: Sindhudurg, Maharashtra |
| 65 | KD19_045_KA-H-2019 | OR179921 | Homo Sapiens | 2019 | India: Karnataka |
| 66 | KD19_0632-KA-H-2019 | OR179922 | Homo Sapiens | 2019 | India: Karnataka |
| 67 | KD19_1857-KA-H-2019 | OR179923 | Homo Sapiens | 2019 | India: Karnataka |
| 68 | KD19_1888_KA-H-2019 | OR179924 | Homo Sapiens | 2019 | India: Karnataka |
| 69 | MCL-20-H-641_MH-H-2020 | OR179915 | Homo Sapiens | 2020 | India: Sindhudurg, Maharashtra |
| 70 | MCL-20-H-690_MH-H-2020 | OR179916 | Homo Sapiens | 2020 | India: Sindhudurg, Maharashtra |
| 71 | MCL-23-H-1303_MBr.P1_RNA11748 | OR179919 | Homo Sapiens | 2022 | India:Shimoga,Tirthalli,Karnataka |
| 72 | MCL-23-H-1305_MBr.P1_RNA11749 | OR179918 | Homo Sapiens | 2022 | India:Shimoga,Honavara,Karnataka |
| 73 | MCL-23-H-1306_MBr.P1_RNA11750 | OR179917 | Homo Sapiens | 2022 | India:Shimoga,Sagara,Karnataka |
| 74 | MCL-23-H-1306CL_RNA11657 | OR179920 | Homo Sapiens | 2022 | India:Shimoga,Sagara,Karnataka |

**Supplementary Table 2**: Representative isolates identified based on the lineages observed in the phylogenetic tree

| **Representative sequence** | **Lineage details** |
| --- | --- |
| P9605_P1-KA-H-1957 | L 1 |
| MG720114_NIV_1722297-KA-H-2017 | L2.2.1 |
| OR162010_MCL-19-H-561-MH-H-2019 | L2.2.2 |
| MG720091_NIV_164187(1297)-KA-H-2016 | L 2.1 |
| OR162020_MCL-19-H-1180-MH-H-2019 | L2.2.2 |
| MG720116_MCL-17-T-296-GA-2017 | L2.2.2 |
| OR179916_MCL-20-H-690-MH-H-2020 | L2.2.2 |
| OR162012_MCL-19-H-553-MH-H-2019 | L2.2.2 |
| OR162018_MCL-19-H-897-MH-H-2019 | L2.2.2 |
| MG720114-NIV_1722297-KA-H-2017 | L2.2.1 |

**Supplementary Table 3: Computational prediction of IL-4 and IL-10 stimulation by short-listed MHC-Class II epitopes belonging to envelope protein of KFDV. Prioritised epitopes for developing vaccine construct are highlighted in yellow.**

| **MHC-Class 2 Epitope** | **IL-4 stimulation** | **IL-10 stimulation** |
| --- | --- | --- |
| AQTVVLELDKTAEHL | **Yes** | **Yes** |
| CGLFGKGSIVACAKF | **Yes** | **Yes** |
| EFGEPHAVKMDIFNL | **Yes** | **No** |
| EKTILTLGDYGDISL | **Yes** | **Yes** |
| GHDTVVMEVTYTGSK | **Yes** | **Yes** |
| GIERLTVVGEHAWDF | **Yes** | **Yes** |
| LPKAWQVHRDWFEDL | **Yes** | **Yes** |
| PGDNIIYVGELSHQW | **Yes** | **Yes** |
| REYCLHAKLANSKVA | **Yes** | **No** |
| RKTASFTTQSEKTIL | **Yes** | **Yes** |
| AVAHGEPNVNVASLI | **No** | **Yes** |
| DQTGILLKSLAGVPV | **No** | **Yes** |
| EGKPSVDVWLDDIHQ | **No** | **Yes** |
| GILLKSLAGVPVANI | **No** | **Yes** |
| SGTQGTTRASLVLEL | **No** | **Yes** |

**Supplementary Table 4**: List of hydrogen and Van der Waal’s interacting residues between TLR2 receptor and vaccine construct. Interactions with epitope regions are in italics and blue text.

| **Hydrogen Bond** | | | | **Van der Waals** | | | |
| --- | --- | --- | --- | --- | --- | --- | --- |
| **TLR2_Residue** | **Vaccine Construct Residue** | **Occupancy** |  | | **TLR2_Residue** | **Vaccine Construct Residue** | **Occupancy** |
| GLN378 | HIE225 | 0.207 |  |  | PRO404 | HIE225 | 0.21 |
| GLN378 | HIE226 | 0.816 |  |  | GLU411 | HIE220 | 0.22 |
| *LYS379* | *TRP117* | *0.222* |  |  | *LYS358* | *GLU96* | *0.22* |
| GLU382 | GLY119 | 0.469 |  |  | *GLN432* | *GLU218* | *0.221* |
| *GLU382* | *HIE115* | *0.935* |  |  | *PHE450* | *THR202* | *0.266* |
| *ARG496* | *ASP207* | *1* |  |  | *ASP405* | *MET198* | *0.274* |
| *LYS358* | *GLU96* | *0.228* |  |  | *LEU472* | *TYR204* | *0.284* |
| LYS353 | GLY118 | 0.386 |  |  | ILE383 | GLY119 | 0.35 |
| LYS353 | GLY120 | 0.584 |  |  | *GLN432* | *PRO219* | *0.36* |
| SER406 | HIE221 | 0.528 |  |  | *SER376* | *TRP117* | *0.37* |
| SER406 | HIE223 | 0.766 |  |  | GLN378 | HIE225 | 0.428 |
|  | | |  |  | *SER471* | *ASP207* | *0.449* |
|  |  |  |  |  | GLU382 | GLY118 | 0.451 |
|  |  |  |  |  | *THR428* | *MET198* | *0.457* |
|  |  |  |  |  | LYS353 | GLY119 | 0.476 |
|  |  |  |  |  | ILE383 | GLY118 | 0.501 |
|  |  |  |  |  | *GLN432* | *VAL217* | *0.503* |
|  |  |  |  |  | GLU382 | GLY119 | 0.539 |
|  |  |  |  |  | *ARG496* | *VAL208* | *0.539* |
|  |  |  |  |  | *GLN378* | *TRP117* | *0.559* |
|  |  |  |  |  | SER406 | HIE225 | 0.563 |
|  |  |  |  |  | LYS353 | GLY118 | 0.592 |
|  |  |  |  |  | CYS407 | HIE221 | 0.602 |
|  |  |  |  |  | THR428 | ALA199 | 0.62 |
|  |  |  |  |  | *SER406* | *VAL169* | *0.626* |
|  |  |  |  |  | LYS353 | GLY120 | 0.628 |
|  |  |  |  |  | SER406 | HIE221 | 0.641 |
|  |  |  |  |  | *SER471* | *VAL205* | *0.685* |
|  |  |  |  |  | GLN408 | HIE224 | 0.702 |
|  |  |  |  |  | *PHE450* | *TYR204* | *0.751* |
|  |  |  |  |  | GLN408 | HIE221 | 0.763 |
|  |  |  |  |  | *LYS379* | *TRP117* | *0.786* |
|  |  |  |  |  | GLN378 | HIE226 | 0.822 |
|  |  |  |  |  | *GLU382* | *HIE115* | *0.928* |
|  |  |  |  |  | SER406 | HIE223 | 0.938 |
|  |  |  |  |  | *SER471* | *TYR204* | *0.948* |
|  |  |  |  |  | *PHE450* | *GLY203* | *0.971* |
|  |  |  |  |  | *ARG496* | *ASP207* | *1* |

**Supplementary Table 5**: List of hydrogen and Van der Waal’s interacting residues between TLR6 receptor and vaccine construct. Interactions with epitope regions are in italics and blue text.

| **Hydrogen Bond** | | |  | **Van der Waals** | | |
| --- | --- | --- | --- | --- | --- | --- |
| **TLR6_Residue** | **Vaccine Construct Residue** | **Occupancy** |  | **TLR6_Residue** | **Vaccine Construct Residue** | **Occupancy** |
| *ASN174* | *THR124* | *0.2* |  | ARG55 | LEU10 | 0.201 |
| PHE101 | ARG74 | 0.207 |  | SER31 | VAL11 | 0.207 |
| *ARG259* | *ALA89* | *0.225* |  | HIE150 | GLY157 | 0.211 |
| GLN283 | GLY93 | 0.262 |  | *VAL284* | *LEU91* | *0.227* |
| *PHE285* | *LEU91* | *0.269* |  | *SER7* | *TYR109* | *0.243* |
| *GLU258* | *LEU91* | *0.278* |  | PHE101 | ARG74 | 0.252 |
| ASN10 | SER66 | 0.288 |  | *ARG259* | *ALA89* | *0.252* |
| THR12 | ARG12 | 0.322 |  | *GLU258* | *THR90* | *0.26* |
| GLU258 | LYS82 | 0.363 |  | GLU258 | GLY92 | 0.262 |
| ASN10 | LEU10 | 0.367 |  | *SER175* | *THR124* | *0.269* |
| ILE151 | LYS77 | 0.372 |  | *HIE53* | *LYS164* | *0.279* |
| SER31 | LEU10 | 0.431 |  | HIE150 | ARG74 | 0.296 |
| SER148 | ARG74 | 0.455 |  | HIE172 | CYS73 | 0.298 |
| ASN10 | ARG12 | 0.491 |  | *ARG55* | *ILE161* | *0.3* |
| ASP102 | ARG74 | 0.578 |  | *PHE285* | *LEU91* | *0.302* |
| ASN10 | CYS65 | 0.635 |  | *GLU258* | *LEU91* | *0.306* |
| HIE172 | GLY156 | 0.67 |  | HIE77 | LYS71 | 0.329 |
| PHE98 | LYS71 | 0.701 |  | GLN283 | GLY92 | 0.334 |
| ASP100 | SER159 | 0.783 |  | GLU258 | LYS82 | 0.352 |
| *ASN174* | *GLN123* | *0.807* |  | GLN29 | SER66 | 0.355 |
| *GLN29* | *LYS164* | *0.846* |  | ARG55 | VAL11 | 0.359 |
| ASP100 | ARG74 | 0.874 |  | SER148 | GLY158 | 0.363 |
| ASN78 | LYS71 | 0.9 |  | *ARG79* | *ILE161* | *0.368* |
| VAL147 | GLY158 | 0.949 |  | *HIE172* | *PHE155* | *0.386* |
| *GLU228* | *THR124* | *0.975* |  | GLN283 | GLY93 | 0.389 |
| ASN8 | SER66 | 0.996 |  | ALA124 | ARG74 | 0.392 |
|  | | |  | *GLN29* | *VAL110* | *0.407* |
|  |  |  |  | TYR149 | GLY157 | 0.408 |
|  |  |  |  | *HIE77* | *LEU162* | *0.428* |
|  |  |  |  | *ASN8* | *VAL110* | *0.445* |
|  |  |  |  | THR12 | VAL11 | 0.447 |
|  |  |  |  | SER31 | LEU10 | 0.484 |
|  |  |  |  | SER148 | ARG74 | 0.485 |
|  |  |  |  | THR12 | ARG12 | 0.486 |
|  |  |  |  | ASN10 | ARG12 | 0.512 |
|  |  |  |  | ASP102 | ARG74 | 0.545 |
|  |  |  |  | ASN10 | VAL13 | 0.576 |
|  |  |  |  | *ASN174* | *PHE155* | *0.609* |
|  |  |  |  | SER148 | GLY157 | 0.613 |
|  |  |  |  | ASN10 | SER66 | 0.617 |
|  |  |  |  | *LYS197* | *VAL125* | *0.631* |
|  |  |  |  | ILE151 | LYS77 | 0.642 |
|  | | |  | *LYS197* | *THR124* | *0.673* |
|  |  |  |  | *PRO173* | *THR124* | *0.733* |
|  |  |  |  | ASN10 | CYS65 | 0.741 |
|  |  |  |  | SER148 | SER159 | 0.768 |
|  |  |  |  | *HIE172* | *GLY156* | *0.784* |
|  |  |  |  | ASN99 | SER159 | 0.816 |
|  |  |  |  | *ASN174* | *GLN123* | *0.842* |
|  |  |  |  | ASN10 | LEU10 | 0.861 |
|  |  |  |  | *HIE53* | *LEU162* | *0.868* |
|  |  |  |  | ASP100 | ARG74 | 0.873 |
|  |  |  |  | VAL147 | GLY157 | 0.895 |
|  |  |  |  | *ASN174* | *THR124* | *0.903* |
|  |  |  |  | ASN8 | CYS65 | 0.906 |
|  |  |  |  | ASP100 | SER159 | 0.959 |
|  |  |  |  | *GLN29* | *LYS164* | *0.972* |
|  |  |  |  | VAL147 | GLY158 | 0.979 |
|  |  |  |  | *GLU228* | *THR124* | *0.995* |
|  |  |  |  | ASN8 | SER66 | 0.998 |
|  |  |  |  | PHE98 | LYS71 | 0.998 |
|  |  |  |  | ASN78 | LYS71 | 1 |
